# The Clinical Effectiveness of Intralesional Injection of 2% Zinc Sulfate Solution in the Treatment of Common Warts

**DOI:** 10.1155/2016/1082979

**Published:** 2016-03-31

**Authors:** Essam-elden Mohamed Mohamed, Khaled Mohamed Tawfik, Asmaa Moneir Mahmoud

**Affiliations:** ^1^Dermatology and Andrology Department, Al Azhar University Hospital, Asyut, Egypt; ^2^Dermatology and Andrology Department, Dermatology Clinic, Asyut, Egypt

## Abstract

*Objective.* To investigate the clinical efficacy and safety of intralesional injection of 2% zinc sulfate solution in the treatment of common warts.* Patients and Methods.* One hundred and twenty patients (78 females and 42 males) aged 5–55 years with 225 common warts participated in this prospective monocentric randomized study. All lesions were treated with intralesional injection of 2% zinc sulfate.* Results.* From 225 warts injected, 135 warts (60%) cured from the first session, 51 warts (22.67%) cured from the second session, and 12 warts (5.33%) cured from the third session. There is no significant relation between improvement and patient's ages, duration, or number of warts (*P* > 0.05). All patients complained from pain during injection, and all treated lesions showed redness, tenderness, and swelling in the first 3 days after injection. Late complications were postinflammatory hyperpigmentation in 90 patients (75%), scaring in 9 patients (7.5%), and ulceration in 3 patients (2.5%). Recurrence occurred in 3 lesions (1.33%).* Conclusion.* The clinical data indicate that intralesional injection of 2% zinc sulfate is an effective maneuver in the treatment of common warts; however, its associated complications limit its use.

## 1. Introduction

Verrucae are benign proliferations seen in skin and mucosae due to infection with papillomaviruses [1]. Although they do not produce acute symptoms and regress spontaneously in two-thirds of lesions within 2 years, they often need treatment due to cosmetic concerns and the dread of autoinoculation [2].

Treatment strategies for warts could be categorized into ablative/cytodestructive (cryotherapy, CO2 laser, trichloroacetic acid, and electrothermal surgery) and topical treatments (podophyllin, podophyllotoxin, imiquimod, cryotherapy, and interferon) [3]. Studies comparing different wart treatments are few [4, 5].

These treatments are considered to be similar in terms of efficacy [5], yet they are different in terms of duration of treatment and cost-effectiveness [6].

Zinc sulfate has an immunomodulatory function and plays a role in enhancing cellular and humeral immunity [7, 8]. Hence, it is used in the treatment of different skin and systemic diseases [9].

Zinc sulfate has been used successfully in the treatment of common warts and genital warts orally [7] and topically in plane warts [10].

The aim of the present work is to assess the clinical efficacy and safety of intralesional 2% zinc sulfate solution in the treatment of common warts.

## 2. Patients and Methods

### 2.1. Recruitment of Patients

A total of 120 patients (42 males and 78 females) with 225 common warts, aged 5 to 55 years, from the attendants of outpatient's clinics in the Department of Dermatology, Al Azhar University Hospital, Asyut, Egypt, between June 2013 and February 2015 were included. The study was approved by the Local Institutional Ethics Committee of Faculty of Medicine, Al Azhar University. The duration of disease ranged from 1 to 24 months (mean ± SD, 7.15 ± 5.04 months). 69 patients had single wart and 51 patients had multiple warts, with mean number of 1.88 ± 1.4, 48 warts. 144 warts (64%) were in the upper limb, 72 warts (32%) in the lower limb, and 9 warts (4%) in the face. All participants were informed about the nature of the study, and written informed consent was obtained.

Exclusion criteria included patients with immunodeficiency, diabetics, history of bleeding tendency or liver disease, and acral warts.

### 2.2. Treatment Protocols

All lesions were treated with intralesional injection of 2% zinc sulfate solution. Zinc sulfate powder was obtained from El-Nasr Pharmaceutical Chemicals Co. (Egypt). The solution was prepared in laboratory at Al Azhar University Hospital, Asyut. In the preparation of 2% zinc sulfate, 2 g zinc sulfate powder was dissolved in 98 mL of sterile distilled water and autoclaved at 95C°. Seventy percent ethanol was used as an antiseptic agent before injection. The injection was given gently until blanching occurs. Assessment of treatment efficacy took place on the basis of clinical examination and photography evaluation using Nikon Coolpix S2500 camera 12 MP.

Follow-up of patients was carried out at two-week intervals to assess clinical response and complication(s) occurred; injection was repeated according to the response to therapy.

### 2.3. Statistical Analysis

The results of the current study were analyzed using a statistical computer package (SPSS version 21). Significant differences between means were evaluated using unpaired independent *t*-test and relations between variables were analyzed using Pearson Chi-Square. *P* values less than 0.05 were considered significant.

## 3. Results

One hundred and twenty patients with 225 common warts were enrolled in this study. 42 patients (35%) were males and 78 patients (65%) were females. The age of the patients ranged from 5 to 55 years with mean ages of 23.6 ± 11.7 years. 69 patients had single wart and 51 patients had multiple warts, with mean number of warts 1.88 ± 1.4. The duration of the lesions ranged from 1 month to 24 months with mean duration 7.15 ± 5.041 months. Sites of injected warts were 144 warts (64%) in the upper limb, 72 warts (32%) in the lower limb, and 9 warts (4%) in the face.

**Figure 1:**
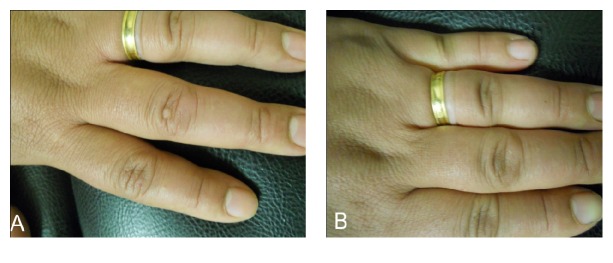
Female patient 34 years old with common wart on the dorsum of middle finger of left hand treated with intralesional injection of 2% zinc sulfate with complete cure (A) before treatment and (B) after treatment.

From 225 warts injected 135 warts (60%) cured from the first session, 51 warts (22.67%) cured from the second session, and 12 warts (5.33%) cured from the third session with a mean number of sessions of 1.63 ± 0.774. From the remaining 27 warts 18 warts (8%) showed moderate response, 3 warts (1.33%) showed mild response, and 6 warts (2.76%) showed no response (Table 1, Figure 1).

There was no significant relation between improvement of warts after treatment and patient's ages, duration of disease, or number of warts (*P* > 0.05) (Table 2).

As regards complications, all patients complained from pain during injection, and all treated lesions showed redness, tenderness, and swelling in the first few days after injection. Late complications were transient postinflammatory hyperpigmentation in 90 patients (75%) which disappeared spontaneously at the end of the study, persistent scaring in 9 patients (7.5%), and ulceration in 3 patients (2.5%). Recurrence occurred in 3 lesions after 6 weeks from the treatment (1.33%) (Table 3).

## 4. Discussion

There is no antiviral treatment that is specific for HPV, but some of the available therapies interfere with the viral life cycle. The most common approach for treatment is to damage or destroy the infected epithelium. This can also induce cell death and antigen exposure and presentation, thereby potentially inducing an immune response [1].

Topical zinc sulfate had been used successfully in the treatment of a wide variety of skin disorders such as cutaneous leishmaniasis [9], leg ulcers [11, 12], recurrent erythema nodosum leprosum [13], perifolliculitis capitis abscedens et suffodiens [14], and alopecia areata [8].

In this prospective study, intralesional injection of 2% zinc sulfate solution was shown to be effective modality in treatment of common warts, as the cure rate was 88%, and most of them (60%) needed just a single injection. Pain during injection was the most common complication. Postinflammatory hyperpigmentation and scaring were the most common late complications. Recurrence of wart at the same site occurred only in three lesions.

Little studies have utilized intralesional injection of 2% zinc sulfate solution for the treatment of common wart. In Sharquine and Al-Nuaimy study [15], out of 173 warts in 53 patients subjected to intralesional 2% zinc sulfate injection, the total number of warts showing complete cure was 170 warts with clearance rate of 98.2% of the treated lesions within 6 weeks of follow-up (80.92% of lesions needed a single injection and showed total clearance within 2 weeks). There are no reported cases of recurrence of wart at the same site previously injected.

The effect of intralesional injection of 2% zinc sulfate was similar or more superior to the effect of topical zinc in previous studies. Topical zinc sulfate (10%) is effective in the treatment of common and plane warts, with 86% complete clearance achieved versus 10% clearance in the placebo group [10]. In double-blinded randomized clinical trials of zinc oxide (20%) versus an salicylic acid/lactic acid ointment, 50% of patients achieved clearance in the zinc oxide group compared with 42% in the salicylic acid/lactic acid group [16].

The mechanism of action of zinc sulfate in viral warts cannot be speculated but is probably similar to the action of zinc sulfate in cutaneous leishmaniasis and bleomycin in viral warts, as both induce necrosis and inflammation [17–20]. When zinc sulfate is injected intradermally, it causes a marked infiltration of inflammatory cells (first a wave of eosinophils then lymphocytes, and finally fibroblasts) towards the injection site [21].

We concluded from this study that intralesional injection of 2% zinc sulfate should be considered as a therapeutic option in the treatment of common warts.

## Figures and Tables

**Table 1:** The clinical effectiveness of intralesional injection of 2% zinc sulfate solution in the treatment of common warts.

Number of sessions	Cumulative period of follow-up	Treated lesions	Cured lesions	Noncured lesions
1st session	2 weeks	225	135 (60%)	90 (40%)
2nd session	4 weeks	90	51 (22.67%)	39 (17.33%)
3rd session	6 weeks	39	12 (5.33%)	27 (12%)
Total number of lesions		225	198 (88%)	27 (12%)

**Table 2:** Relation between improvement of common warts after intralesional injection of 2% zinc sulfate solution and clinical variables.

	Response to treatment	
	*χ* ^2^	*P* value
Age (years)	28.8	0.23
Duration of warts (months)	26.1	0.35
Number of warts	9.7	0.38

Significant (*P* < 0.05).

**Table 3:** Side effects of intralesional injection of 2% zinc sulfate solution in the treatment of common warts.

Complications	Number	%
Early complications (pain, tenderness, or swelling)	120	100%
Late complications		
Postinflammatory hyperpigmentation	90	75%
Scaring	9	7.5%
Ulceration	3	2.5%
